# VCAM1 expression in the myocardium is associated with the risk of heart failure and immune cell infiltration in myocardium

**DOI:** 10.1038/s41598-021-98998-3

**Published:** 2021-09-30

**Authors:** Tongyu Wang, Jiahu Tian, Yuanzhe Jin

**Affiliations:** The Fourth Affiliated Hospital of China Medical University, Yuanzhe Jin, No. 4 Chongshan East Road, Huanggu District, Shenyang, Liaoning Province China

**Keywords:** Mechanisms of disease, Cardiology, Molecular medicine

## Abstract

Ischemic heart disease (IHD) and dilated cardiomyopathy (DCM) are the two most common etiologies of heart failure (HF). Both forms share common characteristics including ventricle dilation in the final stage. Immune mechanisms in HF are increasingly highlighted and have been implicated in the pathogeneses of IHD and DCM. A better understanding of adhesion molecule expression and correlated immune cell infiltration could enhance disease detection and improve therapeutic targets. This study was performed to explore the common mechanisms underlying IHD and DCM. After searching the Gene Expression Omnibus database, we selected the GSE42955, GSE76701, GSE5406, GSE133054 and GSE57338 datasets for different expressed gene (DEGs) selection and new cohort establishment. We use xcell to calculate immune infiltration degree, ssGSEA and GSEA to calculate the pathway and biological enrichment score, consensus cluster to identify the m6A modification pattern, and LASSO regression to make risk predicting model and use new combined cohort to validate the results. The screening stage revealed that vascular cell adhesion molecule 1 (VCAM1) play pivotal roles in regulating DEGs. Subsequent analyses revealed that VCAM1 was differentially expressed in the myocardium and involved in regulating immune cell infiltration. We also found that dysregulated VCAM1 expression was associated with a higher risk of HF by constructing a clinical risk-predicting model. Besides, we also find a connection among the m6A RNA modification ,expression of VCAM1 and immune regulation. Those connection can be linked by the Wnt pathway enrichment alternation. Collectively, our results suggest that VCAM-1 have the potential to be used as a biomarker or therapy target for HF and the m6A modification pattern is associated with the VCAM1 expression and immune regulation.

## Introduction

Heart failure (HF) is a clinical syndrome characterized by fatigue, dyspnea, and fluid retention, commonly caused by left-sided or whole-heart systolic dysfunction and accompanied by congestion^1^. The growth of the aging population and the increased prevalence rates of HF risk factors, including hypertension, diabetes, and obesity, have resulted in an increased prevalence of HF worldwide. A Rotterdam study showed that after adjusting for age, HF patients had a two-fold increased risk of total mortality and a 4–sixfold increased risk of sudden death compared with control subjects^2^.

Ischemic heart disease (IHD) and dilated cardiomyopathy (DCM) are the primary causes of HF. Both syndromes present with clinical manifestations of cardiac insufficiency and overlapping symptoms and signs, but they lack specific manifestations. DCM is typically characterized by nonischemic left ventricular expansion, accompanied by changes in cardiac structure and function, and is the most prevalent cause of chronic congestive HF among individuals between the ages of 20 and 60 years^3,4^. The ventricular structure and function can change due to genetic variations, infections, inflammatory responses, and autoimmune diseases. Therefore, the American Heart Association classifies DCM as inherited, mixed, or acquired based on etiology, with idiopathic and familial diseases representing the most commonly reported causes of DCM^5^. Most HF due to DCM (approximately 70% of DCM-related cases) is attributed to a decrease in the myocardial contractile force caused by ventricular dilatation, whereas IHD causes chronic ventricular remodeling, eventually leading to ventricular dilatation and HF development^6^, suggesting that these two conditions may share a common underlying mechanism that causes HF. In addition to pathological conditions, genetic variations are also known to play roles in the progression of DCM. During recent decades, microarray technology and bioinformatics analyses have been widely used to screen genetic alterations at the genome level, leading to the identification of differentially expressed genes (DEGs) and functional pathways involved in the pathogeneses of many diseases^7^. After searching the Gene Expression Omnibus (GEO), we selected the GSE42955 and GSE57338 gene sets, derived from myocardial array data, for further analysis. The results revealed that vascular cell adhesion molecule 1 (VCAM1) was abnormally expressed in both DCM and IHD patients. Therefore, we speculated that VCAM1 plays an important role in the development of both conditions and could serve as a useful biomarker for prognostic assessments in patients with HF. The goal of this study was to further explore the utility of VCAM1 as a biomarker in HF induced by DCM and IHD.

Studies have implicated chronic inflammation in the development of myocardial structural and functional abnormalities during HF pathogenesis^8^. Inflammatory biomarkers play an important role in the prognostic assessment of patients with HF. For example, Alonso-Martinez et al. showed that patients with acute HF are at increased risk of hospitalization when their C-reactive protein (CRP) levels are > 9 mg/L, and CRP levels have also been associated with HF severity. VCAM1 is an adhesion molecule expressed on the activated endothelial surface, promoting leukocyte adhesion and cross-epithelial migration by binding leukocyte ligands, initiating an inflammatory response^9^. VCAM1 expression levels are significantly increased in patients with HF caused by acute myocardial infarction compared with healthy controls, and VCAM1 levels have good predictive value for patient prognosis^10^. Michowitz et al. showed that VCAM1 mediated the production of reactive oxygen species (ROS) by NADPH oxidase and further activated matrix metalloproteinases to induce ventricular remodeling^11^. The myocardium can be affected by numerous pathophysiological processes that can be broadly classified as ischemic and nonischemic. Ischemic injury is the primary pathophysiological mechanism underlying myocardial injury, and irreversible HF often follows acute ischemic injury or the progressive impairment of cardiac function due to various clinicopathological causes^12^. When the myocardium experiences an ischemic insult, the death of damaged and necrotic cardiomyocytes leads to the activation of tissue-resident immune and non-immune cells. The neutrophil and macrophage populations expand to remove dead cells and matrix debris, leading to the release of cytokines and growth factors that stimulate the formation of highly vascularized granulation tissue (i.e., connective tissue and new vasculature)^13^. The pro-inflammatory cytokines and chemokines produced by immune cells can recruit inflammatory white blood cells from the bloodstream into damaged areas^14^. The immune system drives acute inflammatory and regenerative responses after heart tissue damage^15^, and immune cells are involved in heart damage, ischemia, inflammation, and repair^16^. Although the immune system is known to play an important role in the pathogenesis of heart damage, more research remains necessary to identify the specific underlying mechanisms^17^. This study investigated the influence of *VCAM1* expression on immune infiltration and HF occurrence and assessed the prognostic impact of *VCAM1* expression by building an HF risk prediction model. In addition, we investigated the influence of the N6-methyladenosine (m6A) RNA modification on the expression of *VCAM1* and immune modulation, which has not been explored in-depth.

## Methods

### Acquisition of array data and high-throughput sequencing data

The GSE42955, GSE76701, GSE5406, and GSE57338 gene expression profiles were obtained from the GEO database. The GSE42955 dataset was acquired using the GPL6244 platform (Affymetrix Human Gene 1.0 ST Array [transcript (gene) version]) from a cohort comprised of 29 samples, including heart apex tissue samples from 12 idiopathic DCM patients, 12 IHD patients, and 5 healthy controls. The GSE57338 dataset was acquired using the GPL11532 platform (Affymetrix Human Gene 1.1 ST Array [transcript (gene) version]) from a cohort comprised of 313 cardiac muscle (ventricle tissue) samples obtained from 177 patients with HF (95 IHD patients and 82 idiopathic DCM patients) and 136 healthy controls. The GSE5406 dataset was acquired using the GPL96 platform (Affymetrix Human Genome U133A array) from a cohort containing 210 samples from 16 healthy controls and 194 patients with HF (86 IHD and 108 idiopathic DCM patients). The GSE76701 dataset was acquired using the GPL570 platform (Affymetrix Human Genome U133 Plus array 2.0) from a cohort containing 8 samples obtained from 4 healthy controls and 4 patients with HF (IHD). The raw data in GSE133054, acquired using the GPL18573 platform (Illumina NexSeq 500 [homo sapiens]), was obtained from the GEO database, consisting of samples from a cohort of 8 healthy controls and 7 patients with HF. After acquiring the original data, we annotated the raw data and performed normalization among samples using the SVA package in R. The raw counts from the RNA sequencing (RNA-seq) dataset were transformed into transcripts per million (TPM) to allow for direct comparison of *VCAM1* expression levels. The specific details and raw data can be found in Supplemental Materials.

### DEG screen

We screened DEGs between patients with HF and healthy controls using the limma package in R (limma powers differential expression analyses for RNA-seq and microarray studies). Significance analysis for microarrays was utilized to select significantly different genes with p < 0.05 and log_2_ fold change (FC) ≥ 1. After obtaining DEGs, we generated a volcano plot using the R package ggplot2. We generated a heat map to better demonstrate the relative expression values of specific DEGs across specific samples for further comparisons. The heat map was generated using the ComplexHeatmap package in R (https://jokergoo.github.io/ComplexHeatmap-reference/book/). After the raw RNA-seq data were obtained, the edgeR package was used to normalize the data and screen for DEGs. We used the Wilcoxon method to compare the levels of *VCAM1* expression between the HF group and the normal group.

### Integration of protein–protein interaction (PPI) networks and core functional gene selection

DEGs were mapped onto the Search Tool for the Retrieval of Interacting Genes (STRING) database (version 9.0) to evaluate inter-DEG relationships via protein–protein interaction (PPI) mapping (http://string-db.org). PPI networks were mapped using Cytoscape software, which analyzes the relationships between candidate DEGs that encode proteins found in the cardiac muscles of patients with HF. The cytoHubba plugin was employed to identify core molecules in the PPI network, where were identify as hub genes.

### Establishment of the clinical risk prediction model

The differentially expressed genes showing significant (p < 0.05) correlations with *VCAM1* expression by Spearman’s correlation analysis were further filtered using a least absolute shrinkage and selection operator (LASSO) model. The basic mechanism of a LASSO regression model is to identify a suitable lambda value that can shrink the coefficient of variance to filter out variation. The error plot derived for each lambda value was obtained to identify a suitable model. The entire risk prediction model was based on a logistic regression model. The glmnet package in R was used with the family parameter set to binomial, which is suitable for a logistic model. The cv.glmnet function of the glmnet package was used to identify a suitable lambda value for candidate genes for the establishment of a suitable risk prediction model. The nomogram function in the rms package was used to plot the nomogram. The risk score obtained from the risk prediction model was expressed as:

$$ Riskscore = \sum {\beta \times gene} $$

where β is the value of the coefficient for the selected genes in the risk prediction model and gene represents the normalized expression value of the gene according to the microarray data.

To build a validation cohort, after downloading and processing the data from the gene sets GSE5046, GSE57338, and GSE76701, using the inherit function in R software, we retracted the common genes among the three gene sets, and the ComBat function in the R package SVA was used to remove batch effects.

### Immune and stromal cells analyses

The novel gene signature–based method xCell (http://xCell.ucsf.edu/) was used to investigate 64 immune and stromal cell types using extensive in silico analyses that were also compared with cytometry immunophenotyping^17^. By applying xCell to the microarray data and using the Wilcoxon method to assess variance, the estimated proportions of immune and stromal cell types were obtained for each myocardial tissue sample using a cut-off value of p < 0.05. Cell types were categorized into lymphoid (B cells, CD4^+^ memory T cells, CD4^+^ naive T cells, CD4^+^ T cells, CD4^+^ central memory T cells [Tcm], CD4^+^ effector memory T cells [Tem], CD8^+^ naive T cells, CD8^+^ T cells, CD8^+^ Tcm, CD8^+^ Tem, Class-switched memory B-cells, natural killer [NK] cells, NK T cells [NKT], plasma cells, T helper [Th]1 cells, Th2 cells, T regulatory cells [Tregs], Memory B cells, naive B cells, pro B cells, γδ T cells [Tgd]), myeloid (monocytes, macrophages, macrophage M1, macrophage M2, immature dendritic cells [iDCs], plasmacytoid dendritic cells [pDCs], activated dendritic cells [aDCs], conventional dendritic cells [cDCs], dendritic cells [DCs], neutrophils, eosinophils, mast cells, basophils), stromal (mesenchymal stem cells [MSCs], adipocytes, preadipocytes, fibroblasts, pericytes, microvascular [mv] endothelial cells, endothelial cells, lymphatic endothelial cells, smooth muscle, chondrocytes, osteoblasts, skeletal muscle, myocytes), stem cells (hematopoietic stem cells [HSCs], common lymphoid progenitors [CLPs], common myeloid progenitors [CMPs], granulocyte–macrophage progenitors [GMPs], megakaryocyte-erythroid progenitors [MEPs], multipotent progenitors [MPPs], megakaryocytes, erythrocytes, platelets), and others (epithelial cells, sebocytes, keratinocytes, mesangial cells, hepatocytes, melanocytes, astrocytes, neurons).

### Gene set enrichment analysis (GSEA) and single-sample GSEA (ssGSEA) analysis

To further explore the potential functions of identified genes in HF, samples in the GSE57338 dataset were divided into HF and control groups prior to gene set enrichment analysis (GSEA)^18^. We selected Kyoto Encyclopedia of Genes and Genomes (KEGG) pathways related to immune infiltration that were also associated with the occurrence of HF. We also subdivided the samples according to *VCAM1* expression level (high- and low-expression groups) and performed GSEA for each subgroup. The R package clusterprofiler was utilized to perform the GSEA. The c2.cp.kegg.v7.1.symbols and c5.go.bp.v7.2.symbols gene sets were used as the reference gene sets, and p-adjusted < 0.05 was selected as the cut-off criterion.

To further investigate the pathways that connect m6A modification, immune regulation, and *VCAM1* expression, we used the single-sample GSEA (ssGSEA), which is a specific method for calculating the enrichment scores for pathways in a single sample. We used the GSVA and GSEABase R packages to perform the ssGSEA analysis. The c2.cp.kegg.v7.1.symbols gene set was selected as the reference gene set, and p-value < 0.05, log_2_FC > 1 or log_2_FC <  − 1 were chosen as the cut-off criteria for enriched pathway selection.

### Consensus clustering and analysis of immune parameters among clusters

The expression patterns of 23 m6A regulators identified in the 313 samples contained in gene set GSE57338 were examined using a consensus clustering analysis using a K-means algorithm with Spearman distance, which allowed for the identification of a new gene expression phenotype associated with the occurrence of HF. The analysis was performed using the ConsensusClusterPlus R package, with a maximum cluster number set to 10. The final cluster number was determined by the change in the area under the curve (AUC) for the consensus distribution fraction (CDF) curve.

## Results

### DEGs in the GSE42955 gene set and hub gene selection

**Figure 1:**
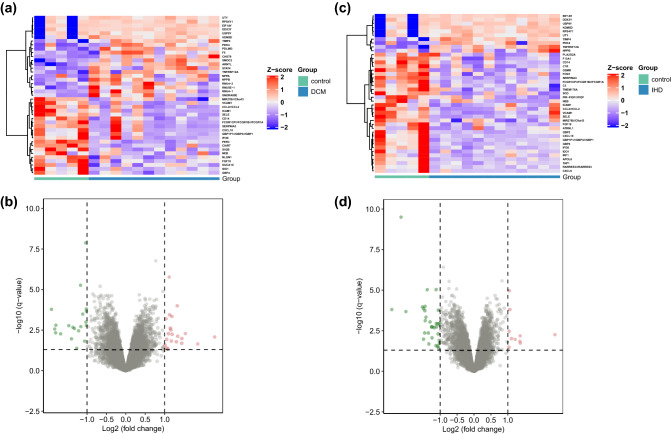

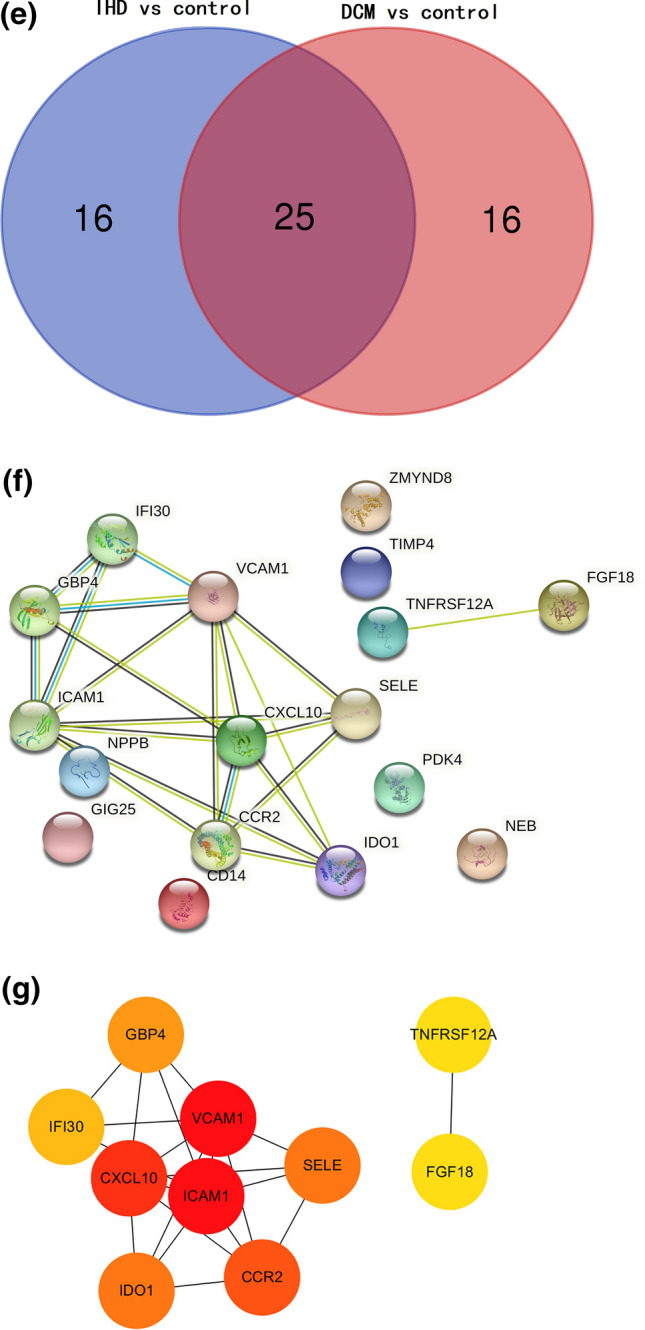
(**a**) Heat map of DEGs screened in myocardial tissue samples of subjects with DCM and controls in the GSE42955 dataset. (**b**) Volcanogram showing DEG screening of myocardial tissue from subjects with DCM and controls in the GSE42955 dataset. (**c**) Heat map of differently expressed genes (DEGs) in myocardial tissue samples of subjects with IHD and controls in the GSE42955 dataset. (**d**) Volcanogram showing DEG screening of myocardial tissue from subjects with IHD and controls in the GSE42955 dataset. (**e**) Intersection of DEGs in the IHD and DCM cohorts. (**f**) Protein–protein interaction (PPI) network for common DEGs. (**g**) The core function modules of the PPI network and the color refers to the connectivity.

The microarray data included in the GSE42955 dataset was divided into two groups (DCM vs. Control and IHD vs. Control) prior to the DEG analysis. With log_2_ FC = 1 as the threshold and p > 0.05 as the standard, 41 DEGs were identified in the DCM vs. Control cohort (21 upregulated and 20 downregulated, Fig. 1a,b), whereas 41 DEGs were selected in the IHD vs. Control cohort (10 upregulated and 31 downregulated, Fig. 1c,d). All the DEGs were shown in Table S1 with detailed p value and log FC. The intersection between the screened genes was identified, and 25 common DEGs were selected (Fig. 1e). The common DEGs were uploaded to the STRING database to form a network of gene interactions (Fig. 1f). The core functional modules were identified using the cytoHubba plugin for Cytoscape software. *VCAM1* and intercellular adhesion molecule 1 (*ICAM1*) had the highest connectivity scores (Fig. 1g).

### Screening DEGs in the GSE57338 dataset and clinical risk prediction model construction

**Figure 2:**
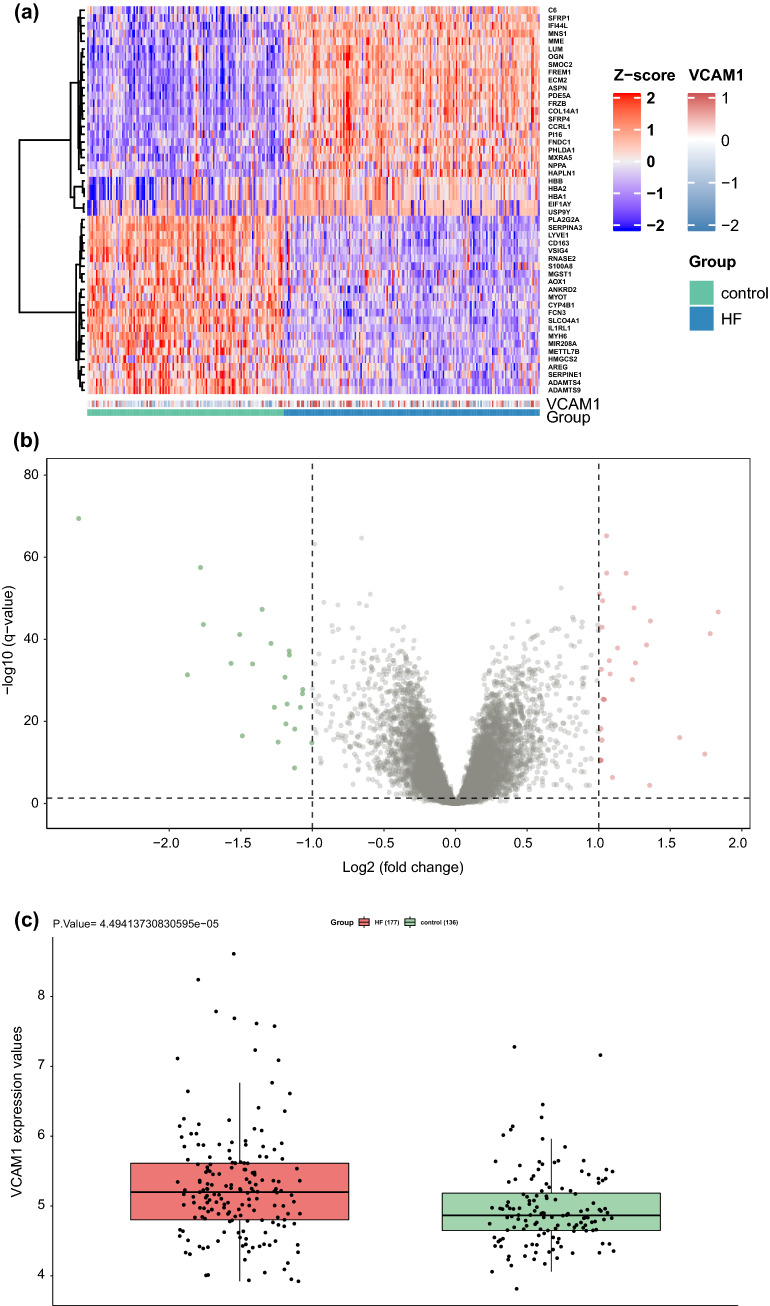

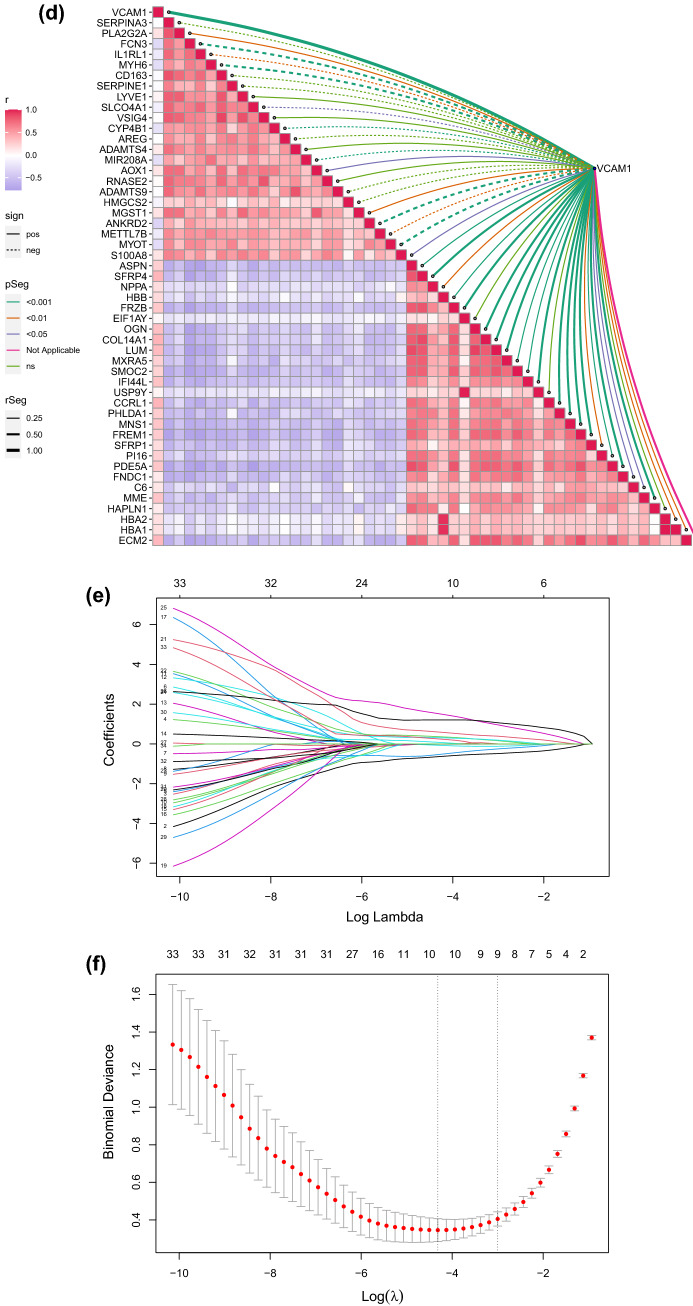

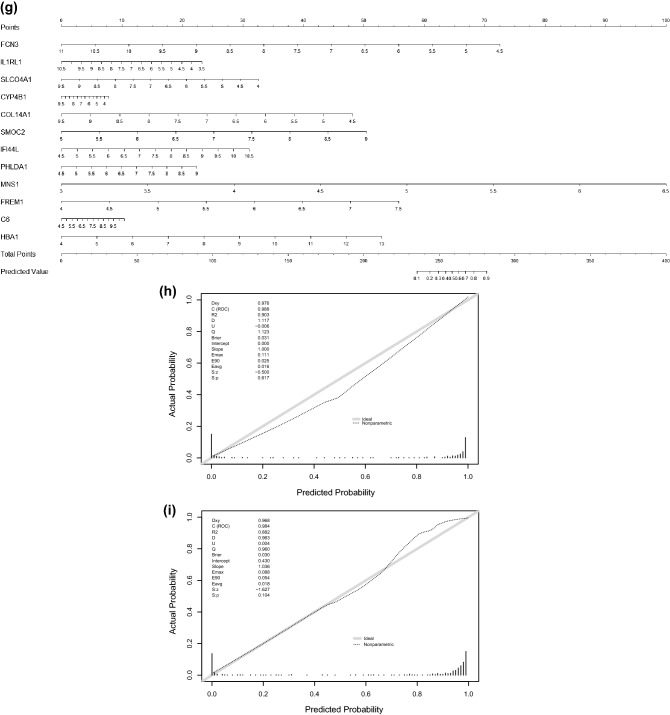

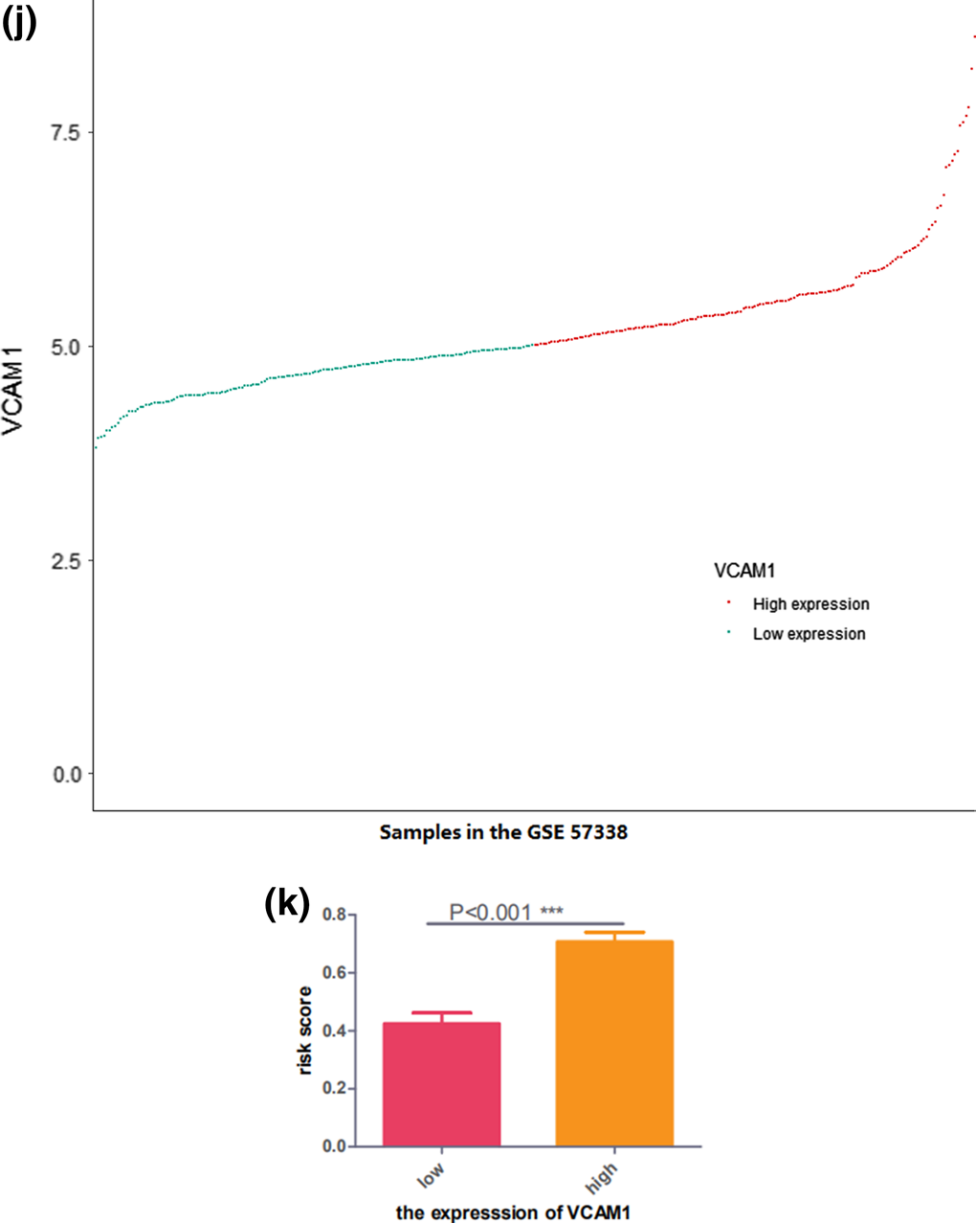
(**a**) Heat map of DEG patterns in myocardial tissue from patients with HF compared with controls in the GSE57338 dataset. (**b**) Volcanogram of DEGs in cardiac tissue from patients with HF compared with controls in the GSE57338 dataset. (**c**) Box plot showing significantly increased VCAM1 gene expression in patients with HF. (**d**) Correlation analysis between VCAM1 gene expression and DEGs. (**e**) LASSO regression was used to select variables suitable for the risk prediction model. (**f**) Cross-validation of errors between regression models corresponding to different lambda values. (**g**) Nomogram of the risk model. (**h**) Calibration curve of the risk prediction model in exercising cohort. (**i**) Calibration curve of predicion model in the validation cohort. (**j**) VCAM1 expression was divided into two groups, and (**k**) risk scores were then compared.

The DEGs in the heart tissue samples from the GSE57338 dataset were identified by comparing the HF group (n = 177) with the non-HF control group (n = 136). A total of 50 DEGs were selected using the thresholds of log_2_FC = 1 and p > 0.05 (Fig. 2a,b). *VCAM1* expression was significantly higher in the HF group, suggesting that *VCAM1* expression may serve as a potential biomarker for HF occurrence and development (Fig. 2c). Spearman’s correlation analysis was subsequently performed on the DEGs identified in the GSE57338 dataset, and 34 DEGs associated with *VCAM1* expression were selected (Fig. 2d) and used to construct a clinical risk prediction model. Variables were screened through the LASSO regression (Fig. 2e,f), and 12 DEGs were finally selected for model construction (Fig. 2g) based on the number of samples containing relevant events that were tenfold the number of variants with lambda = 0.005218785. The Brier score was 0.033 (Fig. 2h), and the final model C index was 0.987. The model showed good degrees of differentiation and calibration. The final risk score was calculated as follows:

Risk score = (− 1.064 × *FCN3*) + (− 0.564 × *SLCO4A1*) + (− 0.316 × *IL1RL1*) + (− 0.124 × *CYP4B1*) + (0.919 × *COL14A1*) + (1.20 × *SMOC2*) + (0.494 × *IFI44L*) + (0.474 × *PHLDA1*) + (2.72 × *MNS1*) + (1.52 × *FREM1*) + (0.164 × *C6*) + (0.561 × *HBA1*).

In addition, a new validation cohort was established by merging the GSE5046, GSE57338, and GSE76701 datasets to validate the effectiveness of the risk model. The principal component analysis (PCA) results before and after the removal of batch effects are shown in Figure S1a and b. The Brier score in the validation cohort was 0.03 (Fig. 2i), and the final model C index was 0.984, which demonstrated that this model has good performance in predicting the risk of HF. We further explored the individual effectiveness of each biomarker included in the risk prediction model. As is shown in Table 1, the effectiveness of *VCAM1* alone for predicting the risk of HF was the lowest, with the smallest AUC of the receiver operating characteristic (ROC) curve. However, the AUC of the overall risk prediction model was higher than the AUC for any individual factor. Thus, this model may serve to complement the risk prediction based on *VCAM1* expression. After a thorough literature search, we found that *HBA1*, *IFI44L*, *C6*, and *CYP4B1* have not been previously associated with HF.

**Table 1:** The effectiveness indicated by the area under curve of ROC operator curve of bio-markers involved in the risk prediction model.

Name of marker	Area under curve of ROC in training cohort	Area under curve of ROC in validation cohort
SMOC2	0.943	0.917
FREM1	0.958	0.937
HBA1	0.687	0.796
SLCO4A1	0.922	0.930
PHLDA1	0.882	0.867
MNS1	0.938	0.883
IL1RL1	0.904	0.928
IFI44L	0.895	0.884
FCN3	0.952	0.953
CYP4B1	0.830	0.829
COL14A1	0.876	0.883
C6	0.788	0.785
VCAM1	0.642	0.663
Effectiveness of risk prediction model	0.988	0.984

Based on *VCAM1* expression levels, the samples from GSE57338 were further divided into high and low *VCAM1* expression groups relative to the median expression level. Comparing the model-predicted risk scores between these two groups revealed that the high-expression *VCAM1* group was associated with an increased risk of developing HF than the low-expression group (Fig. 2j,k).

### Immune infiltration analysis for the GSE57338 dataset

**Figure 3:**
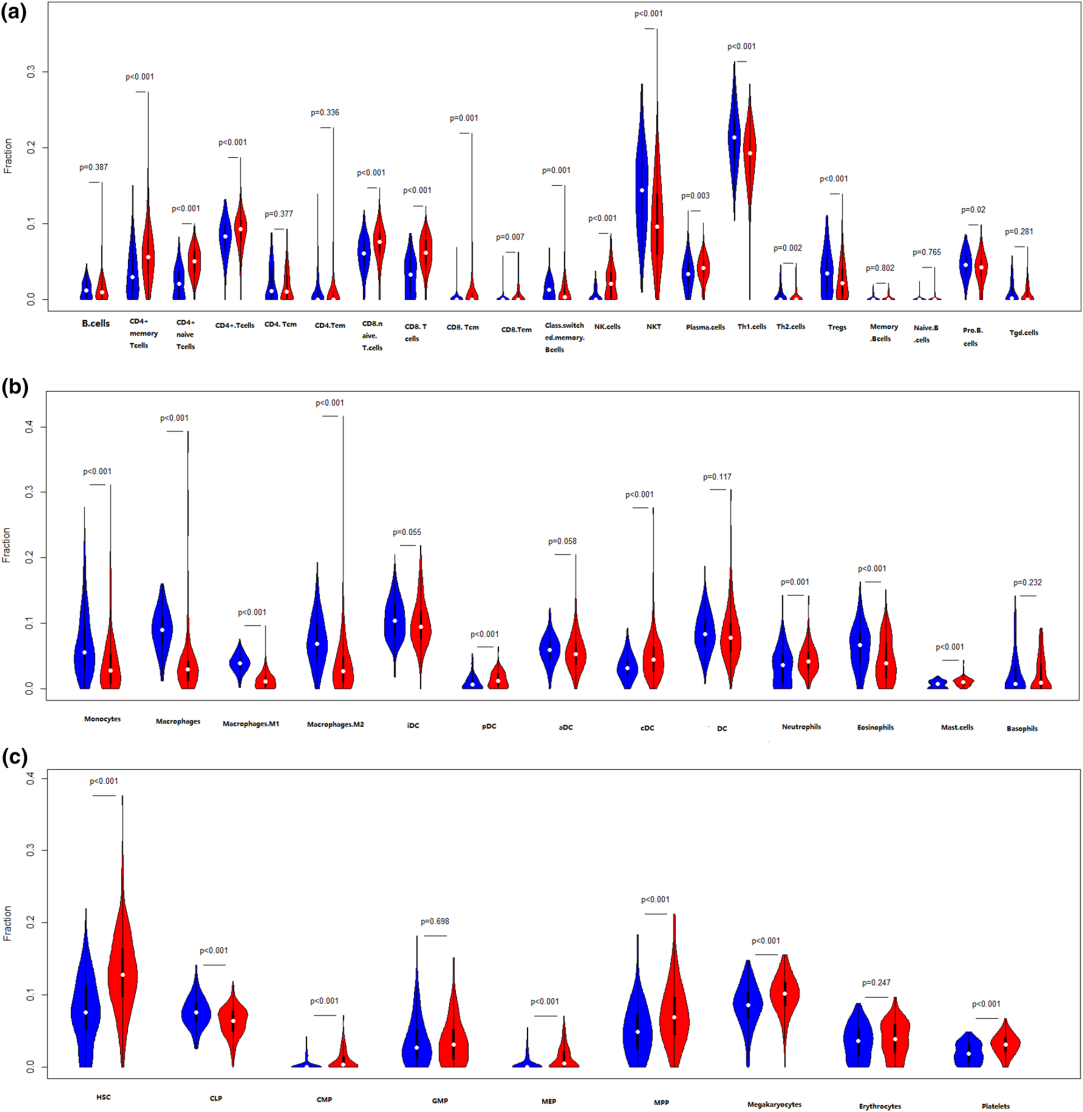

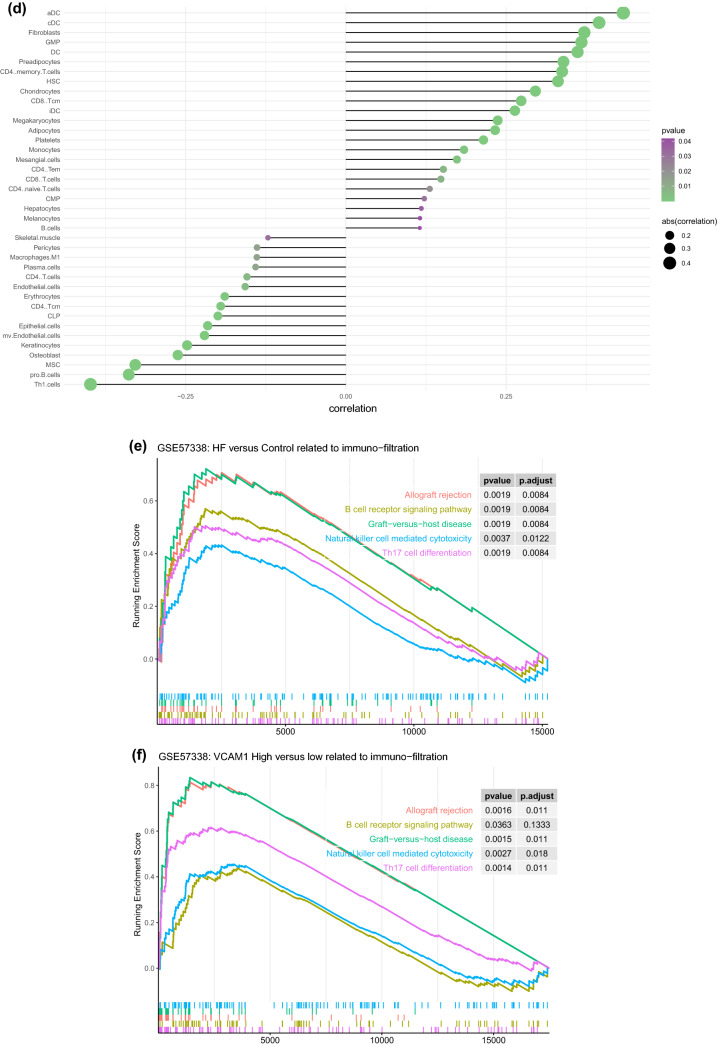

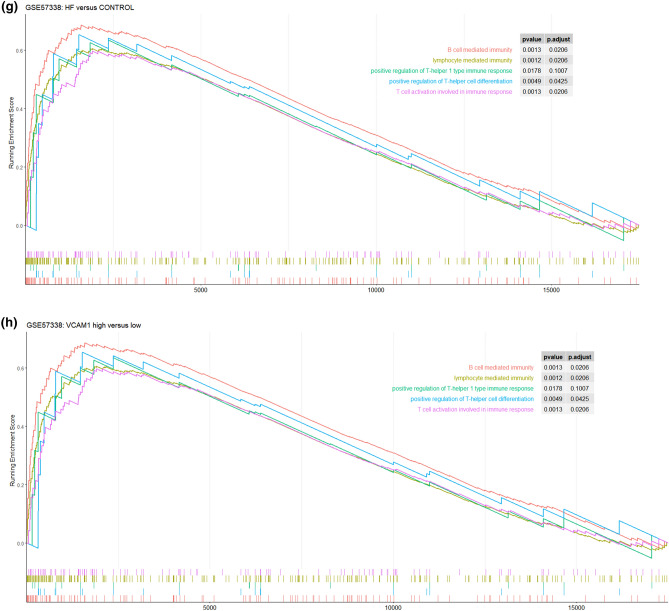

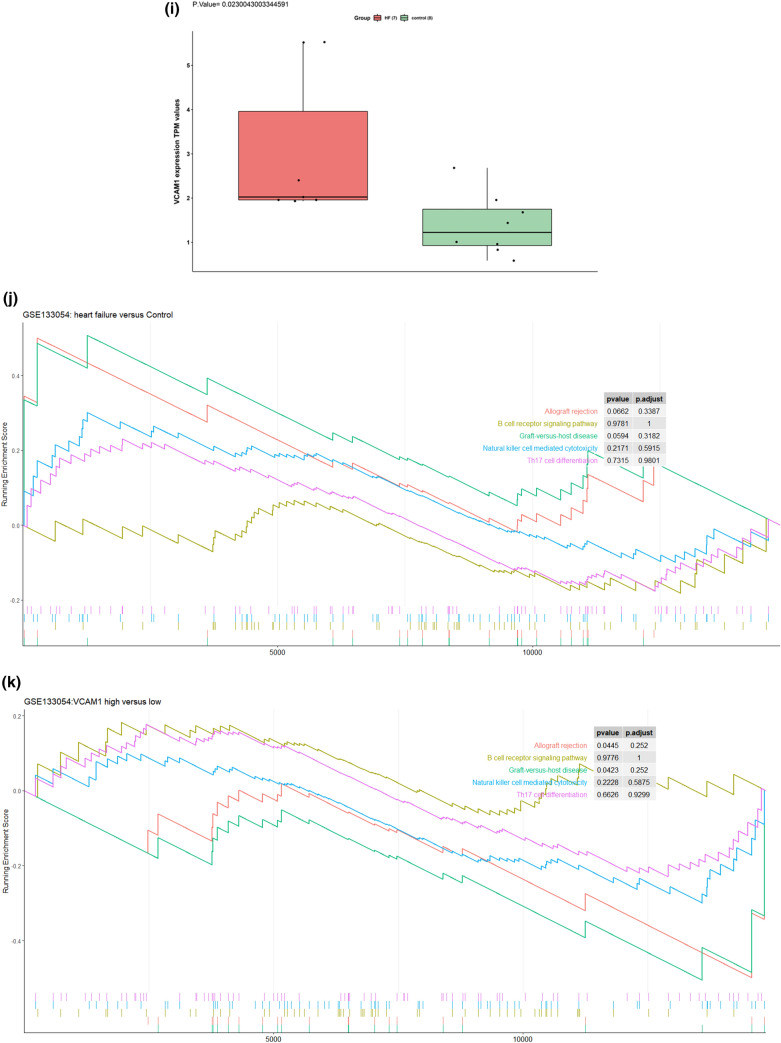

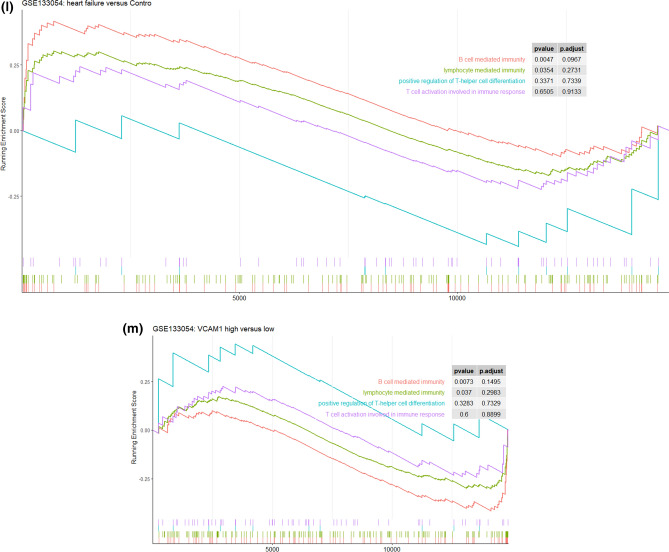
(**a**) The degree of lymphocyte immune infiltration in the HF and control groups (red represents samples from failing hearts and blue represents control samples). (**b**) The degree of myeloid cell immune infiltration in the HF and control groups (red represents samples from failing hearts and blue represents control samples). (**c**) The degree of stem cell immune infiltration in the HF and control groups (red represents samples from failing hearts and blue represents control samples). (**d**) Correlation between VCAM1 expression and the infiltration degrees of various cells. (**e**) GSEA analysis of KEGG pathway enrichment degree between the HF and control groups in GSE57338 gene sets revealed significant difference in the allo-graft rejection, B-cell receptor signaling pathway, Graft versus host diseases natural killer cell mediated cell toxicity and Th17 cell differentiation^57^. (**f**) GSEA analysis of KEGG pathway enrichment degree between the VCAM1 high- and low-expression groups in GSE57338 gene set revealed significant difference in the allo-graft rejection, B-cell receptor signaling pathway, Graft versus host diseases natural killer cell mediated cell toxicity and Th17 cell differentiation^52^. (**g**) GSEA analysis of GO BP enrichment degree between the HF and control groups. (**h**) GSEA analysis of GO BP enrichment degree between the VCAM1 high- and low-expression groups.(**i)** The level of VCAM1 expression in heart failure samples and normal control samples in RNA-seq data-set GSE133054. The result revealed that the level of VCAM1 is significantly higher than control samples. (**j**) The GSEA analysis of KEGG pathway enrichment between the heart failure patients and normal control samples revealed no significant difference in the enrichment of immune related pathways in RNA-seq data-set GSE133054^52^. (**k**) The GSEA analysis of KEGG pathway enrichment between the high VCAM1 expression samples and low VCAM1 expression samples only revealed significant difference in the enrichment of Graft versus host pathway and allograft rejection pathway in RNA-seq data-set GSE133054^52^. (**l**)The GSEA analysis of biological process enrichment between the heart failure patients and normal control samples revealed significant difference in the enrichment of B-cell mediated immunity and lymphocyte mediated immunity in RNA-seq data-set GSE133054. (**m**) The GSEA analysis of biological process enrichment between the high VCAM1 expression samples and low VCAM1 expression samples also revealed significant difference in the enrichment of Graft versus host pathway and allograft rejection pathway in RNA-seq data-set GSE133054.

The immune infiltration analysis was performed on HF and normal myocardial tissue using the xCell database, in which the infiltration degrees of 64 immune-related cell types were analyzed. The results for lymphocyte, myeloid immune cell, and stem cell infiltration are shown in Fig. 3a–c. The infiltration of stromal and other cell types is shown in Figure S2. Most T lymphocyte cells showed a higher degree of infiltration in HF than in normal myocardial tissue, including CD4^+^ memory T cells, CD4^+^ naive T cells, CD4^+^ T cells, CD8^+^ naive T cells, NK cells, and CD8^+^ T cells. The infiltration of myeloid immune cells, including mast cells, cDCs, and pDCs, also showed increasing trends. We subsequently explored the influence of *VCAM1* expression on immune infiltration. As shown in Fig. 3d, *VCAM1* expression positively correlated with Tcm cells, CD4^+^ T cells, CD8^+^ T cells, CD8^+^ naive T cells, cDCs, and CMPs, which were significantly elevated in the HF group relative to the normal group. Conversely, M1 macrophages, myeloid stem cells, and Th1 cells showed negative correlations with *VCAM1* expression, with reduced infiltration in the HF group compared with the normal group. These findings suggest that higher *VCAM1* expression increased the risk of HF by influencing the degree of immune cell infiltration. Using the clusterprofiler package, we explored immune pathway enrichment by performing separate GSEAs in the HF and control groups and in the high and low *VCAM1* expression groups. The HF group showed obvious enrichment of immune infiltration–related pathways (Fig. 3e,f). Subsequent Gene Ontology (GO) Biological Process (BP) enrichment analyses showed the enrichment of BPs related to immune cell activation and differentiation in the high *VCAM1* expression group and in the HF group (Fig. 3g,h). Collectively, these findings indicate that *VCAM1* expression is associated with a higher degree of immune infiltration, which is often associated with an increased risk of HF. To further validate the effects of *VCAM1* expression on the immune infiltration–related pathway and other BPs, we repeated this analysis using an independent RNA-seq gene set (GSE133054). We also identified a significant difference in the *VCAM1* expression levels between patients and healthy controls (Fig. 3i). The subsequent GSEA of the RNA-seq data revealed no significant differences in the immune infiltration–related pathway components between HF patients and healthy controls (Fig. 3j). However, the high *VCAM1* expression group showed significant enrichment in the graft-versus-host pathway and the allograft rejection pathway (Fig. 3k). When examining significant BPs, HF patients were associated with the enrichment of B cell–mediated immunity and lymphocyte-mediated immunity (Fig. 3l), which were also associated with high levels of *VCAM1* expression (Fig. 3m). However, the statistically significant enrichment of the biological process of B-cell mediated immunity and lymphocyte mediated immunity in the RNA-seq results was not maintained when using adjusted p-values.

### The effects of the N6-methyladenosine (m6A)-mediated methylation pattern on immune infiltration and VCAM1 expression

**Figure 4:**
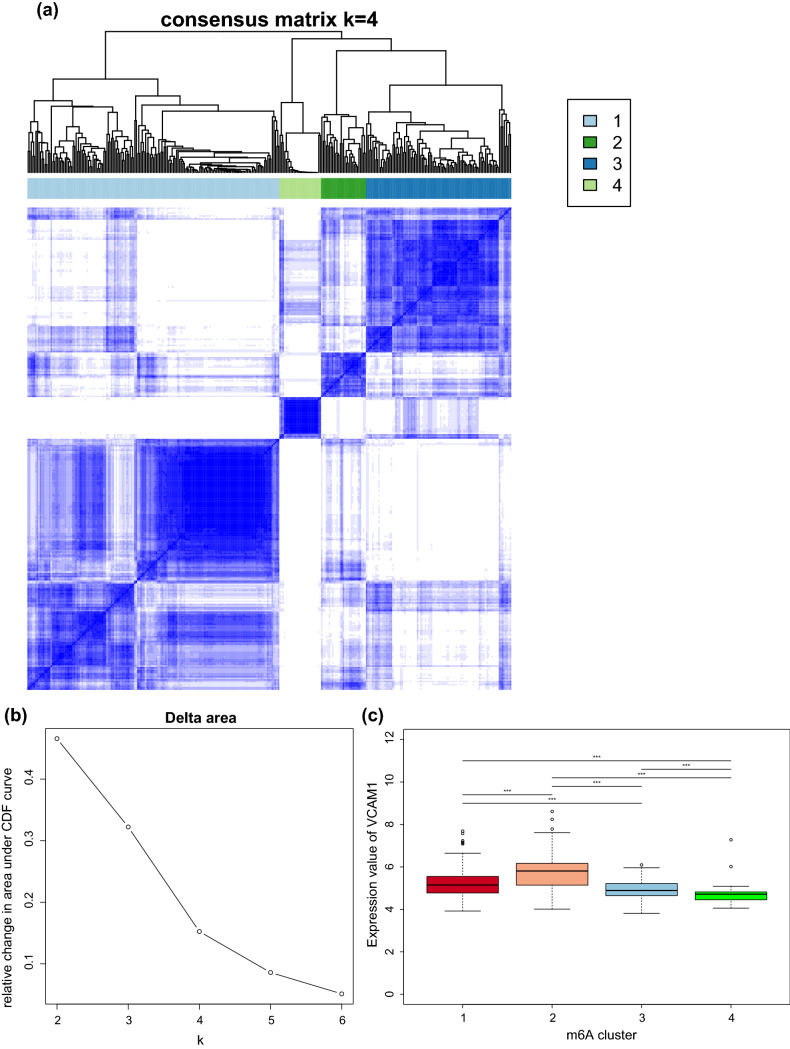

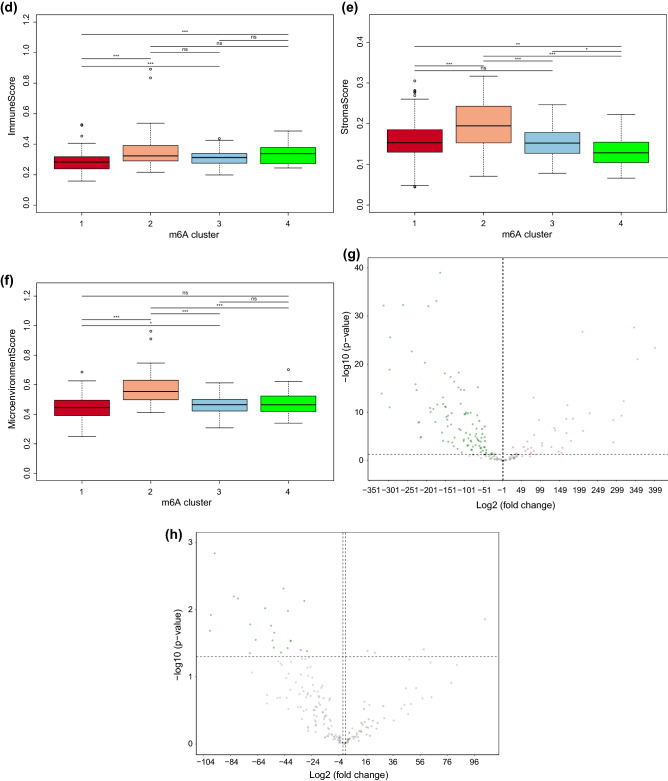
(**a**) Heat-map represents consensus matrix with cluster count of 4. The clusters in the heatmap represents represents the grouping of samples with similar expression patterns of 23 m6A modification regulators. (**b**) The change of area under consensus distribution fraction (CDF) plot. As is shown , when the count of clusters equals to 4 the change of delta area witnessed a turning point which indicate that the heterogeneity within the clusters remained stable. (**c**) The pair wise comparison of the level of VCAM1 across clusters. (**d**) The pair wise comparison of the level of immune score across m6A clusters. (**e**) The pair wise comparison of the level of stroma score across m6A clusters. (**f**) The pair wise comparison of the level of micro-environment score across clusters. (**g**) The subsequent ssGSEA analysis: the volcano plot of comparison of enrichment score between heart failure samples and control samples. There are 36 up regulated pathways and 98 down regulated pathways^52^. (**h**) The subsequent ssGSEA analysis: the volcano plot of comparison of enrichment score between VCAM1 high expression samples and VCAM1 low expression samples. There are 4 up regulated pathways and 22 down regulated pathways^52^.

Recent studies have highlighted the biological significance of the m6A RNA modification in various diseases^19^. However, whether the m6A modifications also play potential roles in the immune regulation of a failing myocardium remains unknown. M6A methylation is a reversible post-transcription modification mediated by m6A regulators, and the pattern of m6A methylation is associated with the expression pattern of the m6A regulators. A total of 23 m6A regulators, including 8 writers (*CBLL1*, *KIAA1429*, *METTL14*, *METTL3*, *RBM15*, *RBM15B*, *WTAP*, and *ZC3H13*), 2 erasers (*ALKBH5* and *FTO*), and 13 readers (*ELAVL1*, *FMR1*, *HNRNPA2B1*, *HNRNPC*, *IGF2BP1*, *IGF2BP2*, *IGF2BP3*, *LRPPRC*, *YTHDC1*, *YTHDC2*, *YTHDF1*, *YTHDF2*, and *YTHDF3*) were identified. We performed a consensus clustering analysis on the 313 samples in GSE57338 to identify distinct m6A modification patterns based on these 23 regulators. Notably, a consensus clustering analysis of the 23 m6A regulators yielded 4 clusters, as shown in Fig. 4a. The reason why the samples were divided into 4 subgroups is that the area under the CDF curve changes most significantly, as shown in Fig. 4b. We explored the relative expression levels of *VCAM1* between the different clusters. Figure 4c shows that *VCAM1* is differentially expressed across m6A clusters. In addition, the immune score, stroma score, and microenvironment score also showed significant differences across different m6A patterns (Fig. 4d–f). We found that cluster 2 was associated with the highest level of *VCAM1* expression and the highest stroma and microenvironment scores. This parallel trend indicated a potential correlation between *VCAM1* expression levels and the regulation of immune infiltration. However, we also found that the immune score, which is an overall evaluation of immune cell infiltration, did not trend in parallel with *VCAM1* expression in the myocardium, which might indicate that the potential regulatory effects of *VCAM1* on the immune microenvironment does not rely completely on immune cell regulation. The pattern of m6A regulators also appears to affect these processes. To further investigate the connections between m6A modification, *VCAM1* expression, and immune infiltration, we utilized the ssGSEA method to calculate pathway enrichment scores in each sample and then identified significant differentially enriched pathways (with threshold: log_2_FC > 1 or < 1 and p-value < 0.05) between HF samples and normal samples and between high and low *VCAM1* expression groups. As shown in Fig. 4g, we identified 134 differentially enriched pathways (including 36 upregulated pathways and 98 downregulated pathways) between HF samples and normal controls. As shown in Fig. 4h and Table S2, we identified 26 differentially enriched pathways (including 4 upregulated pathways and 22 downregulated pathways) between the high and low *VCAM1* expression samples. Of these, 26 pathways overlapped with the pathways described in Table 2. We found that the Wnt signaling pathway was statistically significantly upregulated in HF tissues and high *VCAM1* expresssion objects. The Wnt pathway which was reported linked to multiple steps of HF progression. Thus, we speculated that the m6A regulator expression based RNA modification pattern affected the *VCAM1* expression and subsequently affected the immune cell infiltration via the Wnt signaling pathway.

**Table 2:**
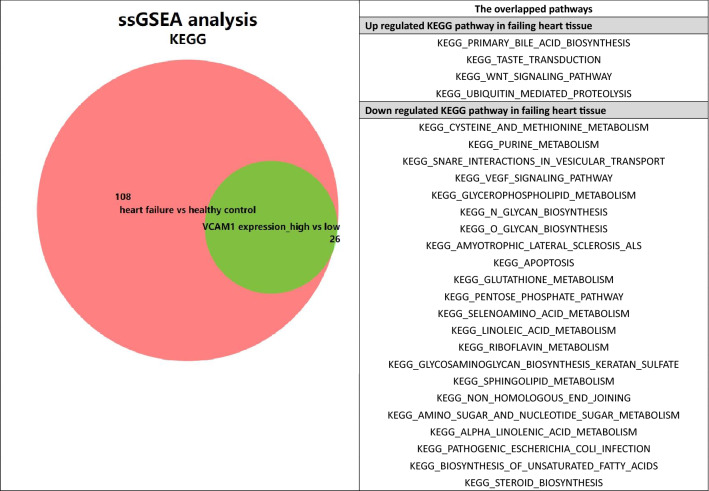
The list of KEGG pathways that is overlapped differently enriched pathways between those grouped by disease state and those grouped by VCAM1 expression value.

## Discussion

HF is a chronic heart syndrome with an average survival time of 5 years after diagnosis, and more than 25 million people are currently at risk of death due to HF worldwide. HF begins with pathological heart remodeling that results in the left ventricle and other cardiac chambers developing progressive structural and functional abnormalities in response to pathological stress^20^. IHD and DCM are two important etiologies associated with HF development^21^. The primary manifestation of HF due to DCM is ventricular enlargement, whereas IHD leads to decreased myocardial cell viability and increased ROS production in response to continuous myocardial ischemia. ROS can directly act on cell membranes and induce myocardial cell apoptosis, resulting in decreased cardiac output. A resulting and gradual increase in cardiac load eventually leads to ventricular remodeling, the final stage of which is ventricular dilation, leading to HF. Although differences in the pathways and factors associated with IHD and DCM and the mechanisms through which they cause HF have been explored^22^, few studies have explored the common pathways and molecules between these two HF etiologies.

This investigation employed bioinformatics methods applied to the GSE42955 and GSE57338 datasets to identify DEGs shared between patients with HF attributed to IHD and DCM. We established an interaction network, which showed that *VCAM1* and *ICAM1* were the genes associated with the highest degrees of connectivity. Previous studies have shown that patients with HF have significantly higher levels of *ICAM1* and *VCAM1* compared with controls, and elevated *VCAM1* expression has previously been associated with HF severity^8^. Therefore, we aimed to explore whether *VCAM1* and *ICAM1* are differentially expressed between HF and normal tissue. An analysis of the myocardial levels of *VCAM1* and *ICAM1* between the HF and control groups in the GSE57338 dataset showed that only *VCAM1* was a significant DEG in this dataset. A correlation analysis between identified DEGs and *VCAM1* expression in the HF group was conducted to identify genes associated with *VCAM1* expression. Finally, we established a risk prediction model using the genes identified as correlating with *VCAM1* expression. The subsequent analysis showed that the risk of HF increased with higher *VCAM1* levels.

VCAM1 is an adhesion molecule found on the endothelial surface that enhances binding with white blood cells, increasing leukocyte adhesion and epithelial cell migration^23^. Experimental studies have shown that immune response mechanisms correlate with pathological heart remodeling, causing left ventricular dysfunction and eventually leading to HF. Therefore, we explored the relationship between VCAM1, the myocardial infiltration of immune cells, and subsequent effects on HF risk^24^. The xCell algorithm was used to predict the degree of infiltration for various immune cells in cardiac tissue, and correlation analysis was conducted to assess the relationship between *VCAM1* expression and the degree of infiltration for various immune cells. The results showed that the *VCAM1* expression level was positively correlated with the numbers of CD8^+^ T cells, CD8^+^ Tcm cells, CD4^+^ naive T cells, cDCs, CMPs, and other immune cells, and these cells also displayed a higher degree of infiltration in HF tissue than in normal tissue. Previous studies have shown that monocytes that infiltrate the myocardium can differentiate into macrophages and promote tissue damage repair^25^. As highly specific antigen-presenting cells involved in adaptive and innate immunity, DCs also play important roles in the occurrence of HF. Animal experiments revealed that exogenous DCs induced autoimmune inflammation, mediated by CD4^+^ T cells, promoting ventricular dilation and HF^26^. Increased T lymphocyte infiltration, which is involved in adaptive immunity, was also associated with increased HF risk^27^. One of the most important features of chronic HF is the presence of numerous mature T cell infiltrates in the myocardial tissue^28,29^. Animal studies have shown that T cell–deficient mice are less likely to develop HF after aortic ligation^30^, and the alternation of T cell subsets promotes HF development, as indicated by elevated brain natriuretic peptide levels^31^. In vitro experiments revealed that Th1 cells—an important subset of T cells—can release interferon-α to stimulate the transformation of myocardial fibroblasts into γ-smooth muscle actin fibroblasts, which can promote myocardial fibrosis, an important ventricular remodeling process^32^. Therefore, T cells and their subsets play important roles in HF occurrence and pathogenesis^33^. Myeloid immune cells are the most abundant immune cells in the myocardium. Immune cells in healthy subjects do not produce harmful chronic inflammation under physiological conditions, but under pathological conditions, such as acute or chronic ischemia, the degree of myeloid immune cell infiltration in the myocardium increases, resulting in the release a variety of inflammatory mediators that stimulate chronic fibrosis and remodeling, exacerbating HF^34^. The results of this study revealed an increase in the degree of infiltration by myeloid progenitors and cells in HF tissues that positively correlated with *VCAM1* expression, which can stimulate the differentiation of myeloid progenitors into macrophages and monocytes. An uncontrolled inflammatory response during the pathological state triggers a large number of monocytes to differentiate into macrophages, causing tissue damage, and extensive monocyte infiltration in cardiac tissue has been associated with an increased risk of HF^35^. Most immune cells are recruited from the blood, and as an adhesion factor expressed on the vascular endothelium, *VCAM1* can recruit myeloid progenitor cells to infiltrate the myocardium, where they differentiate into various subsets of myeloid immune cells, promoting HF^36^. In our study, *VCAM1* expression was positively correlated with immune cells infiltration, leading to our hypothesis that the increased risk of HF associated with elevated *VCAM1* expression is due to the VCAM1 regulation of immune cell infiltration.

We also conducted a GSEA to examine immune infiltration–related KEGG pathways, comparing between HF and normal tissues and between high and low *VCAM1* expression groups. The results showed that immune-related pathways were enriched in both HF tissues and in tissues with high *VCAM1* expression, including signaling pathways associated with the graft-versus-host response and Th17 differentiation. The proportion of Th17 cells in the blood circulation and the level of cytokine secretion increase in patients with HF^37^. In addition, the differentiation of Th17 cells often requires transforming growth factor-β and interleukin (IL)-6, which are involved in myocardial fibrosis development. IL-23, which is secreted by Th17 cells, promotes the secretion of granulocyte–macrophage colony-stimulating factor by Th17 cells, the infiltration of other immune cells, and the development of a chronic inflammatory response^38^. An increase in Th17 cells is often accompanied by a decrease in Treg cells^39^, which is consistent with the results observed in this study. Therefore, we propose that the elevated HF risk associated with *VCAM1* expression is mediated by Th17 cell infiltration. We also observed that autoimmune-related graft-versus-host and xenograft rejection pathways were significantly enriched in the myocardial tissues of patients with HF and subjects with increased *VCAM1* expression, supporting the autoimmune response as important mechanisms for HF occurrence and development^40^. B cell pathways were also enriched in HF tissues and in myocardial tissue with increased *VCAM1* expression, and B cell activation has been associated with the production of autoimmune antibodies^41^. Cytotoxic pathways found in NK cells that play roles in graft immune rejection and cause cell damage through direct contact with graft cells^42^ were also enriched in our results. Based on our observation of increased NK cell infiltration in the myocardial tissues of patients with HF, *VCAM1* expression may regulate NK cell–mediated cytotoxicity, promoting myocardial injury by participating in related signaling pathways. In addition, GSEA revealed that functions associated with T and B cell activation were enriched in HF patients and in subjects with high *VCAM1* expression, supporting a role for *VCAM1* in the regulation of immune cell infiltration in HF. We validated our GSEA findings in an RNA-seq gene set. Although the results in the novel gene set demonstrated the enrichment of pathways related to immune reactions (including allograft rejection, B cell receptor pathway, graft-versus-host reaction, NK cell–mediated cytotoxicity, and Th17 cell differentiation), these differences did not reach the level of significance between HF and normal control samples. In individuals with high *VCAM1* expression levels, the significant enrichment of pathways related to allograft rejection and graft-versus-host reaction was observed. In the GSEA BP analysis, we found that B cell–mediated immunity and lymphocyte-mediated immunity were significantly different between HF and col samples. A similar trend was observed comparing samples with high and low levels of *VCAM1*. This difference between the microarray and RNA-seq results may be due to the relatively small number of samples examined by RNA-seq compared with the number of samples analyzed by microarray, in addition to differences in sensitivity between these methods. However, these findings still indicate that the differential expression of *VCAM1* influences pathways and biological responses associated with immune reactions.

We also established a risk model for HF using the differently expressed genes identified between HF and normal control tissue that were correlated with *VCAM1* expression. The final risk prediction analysis showed good performance in both the training and validation cohorts. Previous studies reported biomarkers, such as ficolin 3 (FCN3), are associated with the progression of HF^43^. IL-1–like receptor 1 (ILRL1), also known as ST2 protein, represents a promising target for HF therapy and is actively involved in T cell–mediated immune responses^44^. In animal studies, the lack of collagen type XIV alpha 1 chain (COL14A1) promotes pressure overload, resulting in myocardial hypertrophy, a critical step in the progression of HF^45^. Previous studies identified SPARC-related modular calcium-binding protein 2 (SMOC2) as a dysregulated component of the inflammatory pathway following the analysis of tissue associated with right ventricular failure (RVF)^46^. Pleckstrin homology–like domain family A member 1 (PHLDA1) is a new target for oxidative stress and ischemia-perfusion–induced myocardial injury^47^. These traditional biomarkers have demonstrated good performance in predicting the risk of HF in our training and validation cohorts. Meiosis-specific nuclear structural 1 (MNS1), solute carrier organic anion transporter family member 4A1 (SLCO4A1), and FRAS1-related extracellular matrix 1 (FREM1) were included in a risk prediction model established by the support vector machine method. However, that model was not validated in a new cohort^48^. We also investigated the performance of the individual biomarkers included in the prediction model. After searching the literature, we found that hemoglobin subunit alpha 1 (HBA1), interferon-induced protein 44–like (IFI44L), complement component 6 (C6), and cytochrome P450 family 4 subfamily B member 1 (CYP4B1) have not previously been reported in association with HF. Therefore, the newly defined model could be applied clinically to predict HF risk. Although, we found that *VCAM1* expression had the lowest HF risk predictive ability, the developed risk prediction model can serve as a complementary method for integrating novel and traditional biomarkers, magnifying the utility of these biomarkers in the prediction of HF risk. Few studies have examine HF therapies that target VCAM1, and our results may provide evidence for future treatments.

Emerging evidence has demonstrated that the m6A post-transcriptional RNA modification plays an essential role in innate immunity and inflammatory reactions, mediated by diverse m6A regulators, which modify m6A patterns^49^. Although several elegant studies have revealed the epigenetic modulation mediated by m6A regulators in the immune context, the immune characteristics in the myocardium associated with varying m6A modification patterns have not yet been investigated. Therefore, identifying distinct immune characteristics and the value of *VCAM1* by examining associations with the m6A pattern can help us further understand the regulation of *VCAM1* expression and its association with immune mechanisms in the development of HF. Our results showed that the *VCAM1* expression value, the immune score, the microenvironment score, and the stroma score were significantly different across different patterns of m6A modifications. Cluster 2 was associated with the highest *VCAM1* expression level compared with the other clusters. The immune microenvironment and stroma scores were also higher in cluster 2 than in other clusters. Thus, we speculated that *VCAM1* expression is regulated by m6A modifications, and *VCAM1* is involved in the modulation of the immune microenvironment, as the microenvironment score showed parallel trends with *VCAM1* expression across the different patterns of m6A modifications. We also found that alternations in the stroma score resembled changes in *VCAM1* level across the different m6A patterns. These findings suggest that *VCAM1* regulates the immune microenvironment primarily by regulating immune stromal cell infiltration. We also investigated the pathways connecting *VCAM1* with immune regulation and found that the Wnt signaling pathway is upregulated in both HF samples and those with high *VCAM1* expression. As previously reported, the Wnt signaling pathway participates in multiple steps of HF progression, including cardiomyocyte apoptosis, cardiac fibrosis, angiogenesis, and inflammation^50^. We found that the changes in *VCAM1* expression levels alter the enrichment of the Wnt signaling pathway. Thus, we speculate that *VCAM1* regulates the activation of the Wnt signaling pathway, leading to the modulation of the inflammatory response and immune microenvironment and promoting the clearance of cellular debris created during myocardial infarction–induced cellular apoptosis, a common cause of HF^51^.

### Limitations

This study established a predictive model according to the biomarkers showing statistically significance with *VCAM1* using Spearman correlation method. However, our STRING database search revealed that *VCAM1* does not directly interact with any of the selected biomarkers used for the risk prediction model. Thus, our research only reveals a correlation in expression values, with no indication of the functional mechanism underlying these correlations. The model was used to calculate risk scores for each sample and examine differences between high and low *VCAM1* expression. Although studies have investigated the association between *VCAM1* and HF, most have focused on circulating *VCAM1* levels. For example, in the MESA cohort, over a median follow‐up of 14.4 years, researchers found that higher serum *VCAM1* levels were associated with progressively increased risks of HF and HF with preserved ejection fraction (HFpEF)^52^. A study involving 120 chronic HF patients and 69 healthy controls found that circulating *VCAM1* served as an independent mortality predictor^53^. However, circulating *VCAM1* can be affected by comorbidities, such as immunological diseases, cancer, and autoimmune myocarditis. Thus, using circulating *VCAM1* as a predictor of HF incidence may be biased, and circulating *VCAM1* measurements require standardization and validation in clinical settings^54^.

Previous studies of immune cell contributions to HF only investigated the differences in CD34^+^ stem cell populations among DCM patients, IHD patients, and healthy controls. In our study, the relationship between *VCAM1*, an important endothelial adhesion molecule, and immune cell infiltration in the myocardium was explored^55^.

We did not examine the role of high *VCAM1* expression levels in healthy samples. A prospective cohort study is more suitable for exploring the long-term effects of increased *VCAM1* expression in a healthy population. Based on the comparison of risk scores between high and low *VCAM1* expression groups, we conclude that healthy control populations with higher *VCAM1* expression are at increased risk of HF if they experience an event that contributes to HF; however, the current case–control retrospective study is not suitable for drawing such conclusions. The enrichment analysis of RNA-seq data revealed an unstable pattern due to the limited number of samples. However, the current p-values were sufficiently significant to indicate an effect of dysregulated *VCAM1* expression on immune-related pathways. However, this study only involved gene sets examining idiopathic DCM, and the potential for *VCAM1* to serve as a predictive marker for familial DCM or to differentiate familial from idiopathic DCM was not investigated. Future studies can further investigate the ability of *VCAM1* to differentiate the underlying etiology of DCM across multiple levels, as different types of DCM are associated with different prognosis^56^.

## Conclusions

*VCAM1* can be considered a useful biomarker for identifying individuals at high risk of HF. The protein likely acts through the regulation or participation in the recruitment of immune cells to the site of heart injury or repair. We established a clinical risk prediction model involving DEGs correlated with *VCAM1* expression to evaluate the risk for HF and complement *VCAM1* levels in the prediction of HF risk. In addition, we explored 4 patterns of m6A modifications based on the expression of 23 m6A regulators and investigated the effects of different m6A modification patterns on the expression of *VCAM1* and immune cell infiltration in heart tissue. The results revealed that both *VCAM1* expression and the immune cell infiltration pattern were associated with the m6A modification pattern. We also found that the immune stroma score and microenvironment score moved in parallel trends across the different m6A modification patterns, which may be associated with the upregulation of the Wnt pathway in response to changes in *VCAM1* expression. The subsequent ssGSEA analysis revealed that the Wnt signaling pathway might connect *VCAM1* to immune modulation.

## Supplementary Information

Supplementary Information 1.

Supplementary Information 2.

Supplementary Information 3.

Supplementary Information 4.

Supplementary Information 5.

## Data Availability

We provide the raw data and raw codes in Supplementary files.
