# Supplementary figures and images for: VCAM1 expression in the myocardium is associated with the risk of heart failure and immune cell infiltration in myocardium
Source: Sci Rep. 2021 Sep 30;11:19488. doi: 10.1038/s41598-021-98998-3 (PMC8484263; doi:10.1038/s41598-021-98998-3)

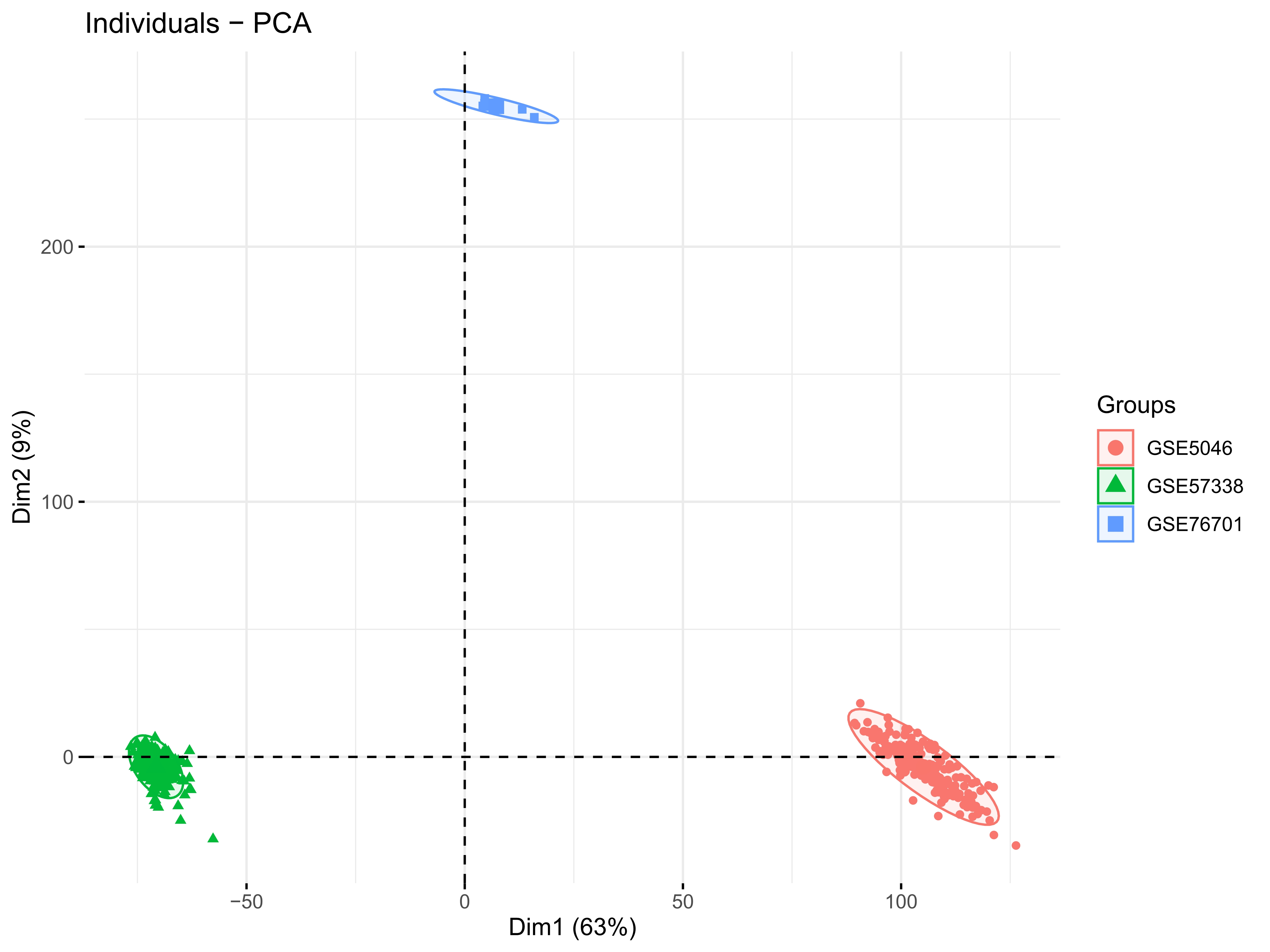

Supplement: Supplementary file 1 — Supplementary Information 1. [file 41598_2021_98998_MOESM1_ESM.jpg]

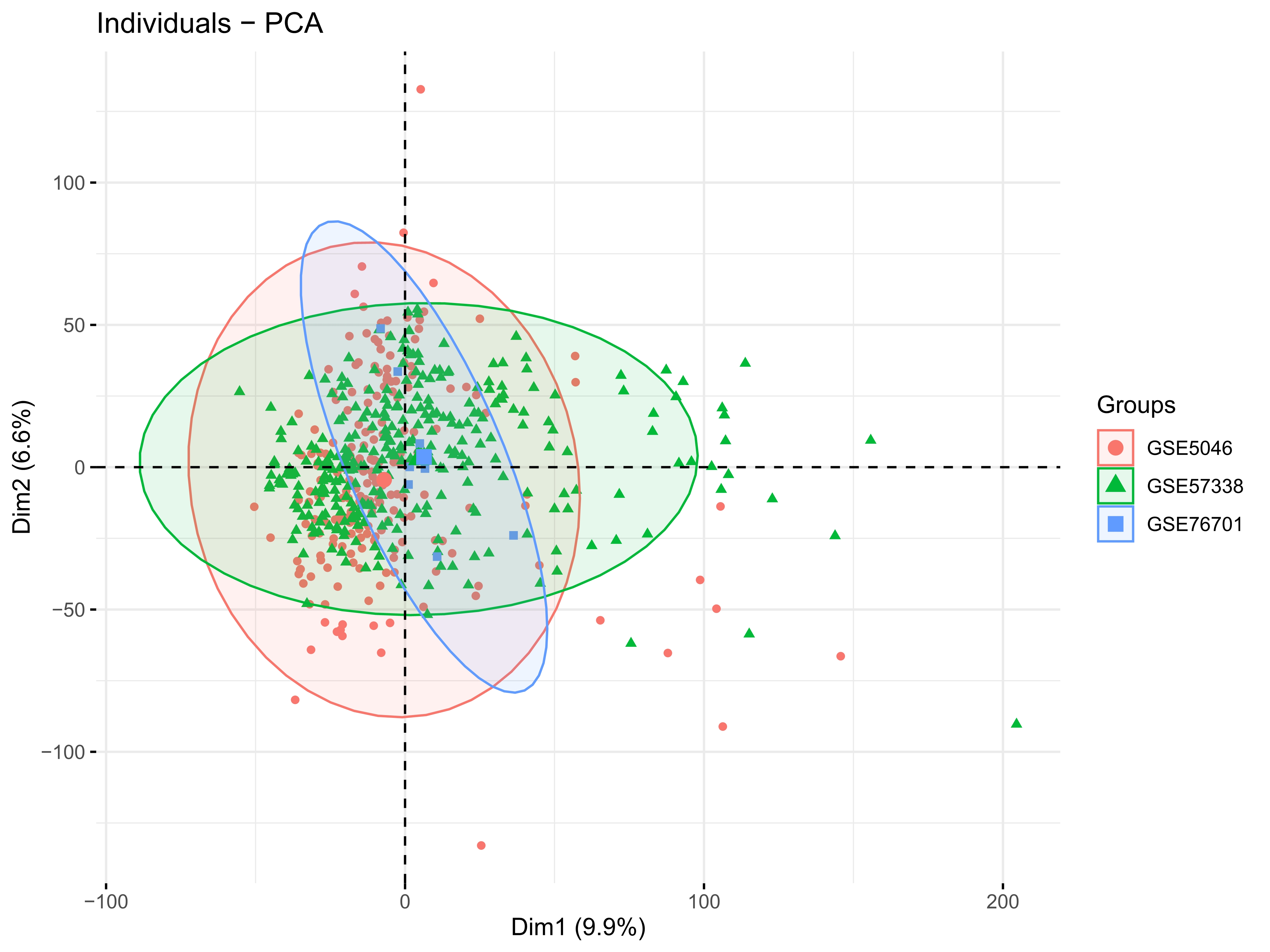

Supplement: Supplementary file 2 — Supplementary Information 2. [file 41598_2021_98998_MOESM2_ESM.jpg]

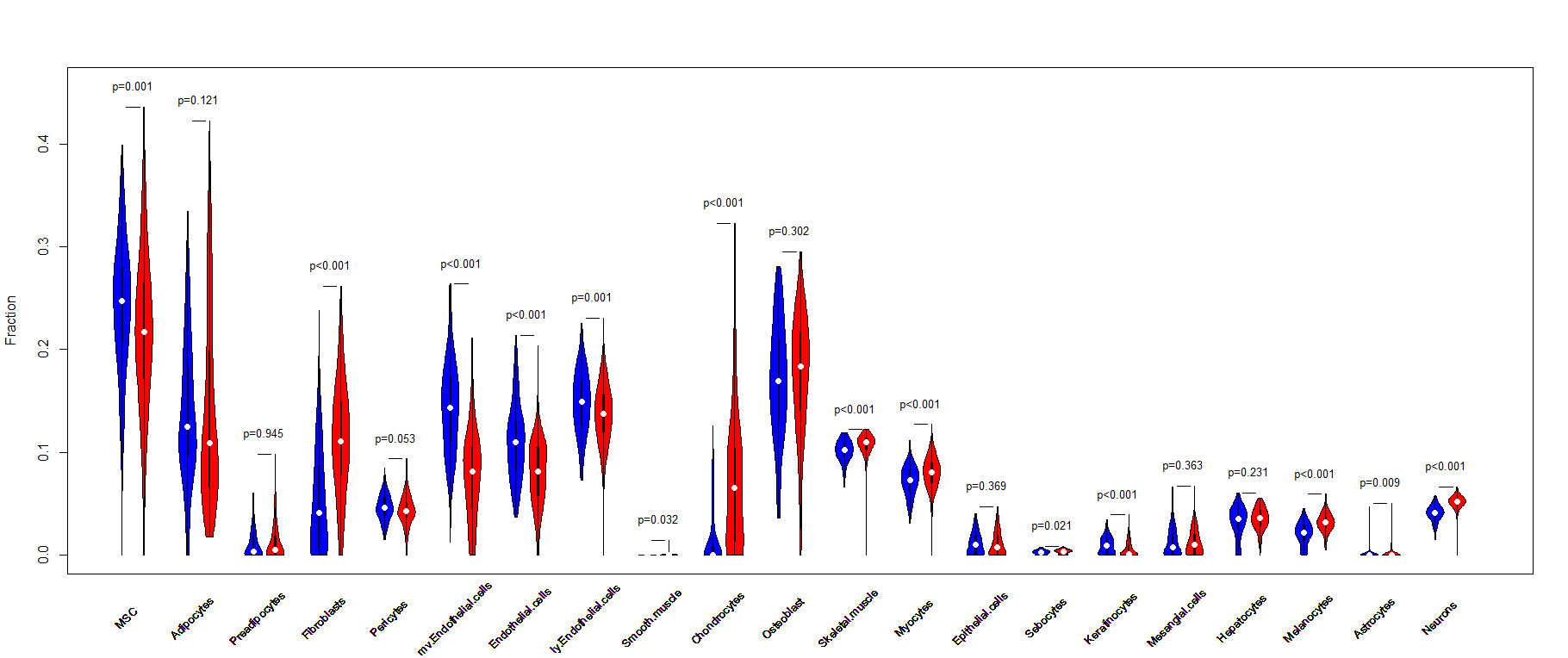

Supplement: Supplementary file 3 — Supplementary Information 3. [file 41598_2021_98998_MOESM3_ESM.png]
