# Supplementary material for: VCAM1 expression in the myocardium is associated with the risk of heart failure and immune cell infiltration in myocardium
Source: Sci Rep. 2021 Sep 30;11:19488. doi: 10.1038/s41598-021-98998-3 (PMC8484263; doi:10.1038/s41598-021-98998-3)
Supplement: Supplementary file 4 — Supplementary Information 4. [file 41598_2021_98998_MOESM4_ESM.docx]

|  | IHD vs control | | DCM vs Control | |
| --- | --- | --- | --- | --- |
|  | LogFC | P.value | LogFC | P.value |
| SELE | -2.013597989 | 0.000208454 | -1.915661552 | 0.000166598 |
| SERPINA3 | -2.157114529 | 3.16E-10 | -1.80965233 | 0.004627067 |
| CCL2 | -1.453717 | 0.004341875 | -1.802699816 | 0.001583757 |
| CXCL10 | -2.431014115 | 0.000155867 | -1.724728224 | 0.007097833 |
| IDO1 | -1.489006052 | 0.010375928 | -1.680025506 | 0.005362413 |
| CD14 | -1.380594937 | 9.46E-06 | -1.470513055 | 0.001686523 |
| FCGR1CP | -1.526226793 | 0.000108203 | -1.400984624 | 0.010908085 |
| FGF18 | -1.439993807 | 0.000770844 | -1.363676293 | 0.002140072 |
| MIR27B | -1.344861563 | 0.004352737 | -1.325875213 | 0.002522297 |
| IFI30 | -1.441972477 | 0.000100645 | -1.266941586 | 0.041690063 |
| GBP4 | -1.488441224 | 0.000139472 | -1.216612029 | 0.0034991 |
| ICAM1 | -1.023430741 | 0.018961289 | -1.1603835 | 0.00191953 |
| GBP1P1 | -1.44505923 | 0.000189206 | -1.061951514 | 0.015195199 |
| NEB | -1.331384799 | 0.020532565 | -1.036657141 | 0.007478951 |
| VCAM1 | -1.104535083 | 0.001913426 | -1.026265213 | 0.001017797 |
| EIF1AY | 2.392401356 | 0.005522383 | 2.29181423 | 0.008384175 |
| NPPB | 1.236968902 | 0.093026671 | 1.856782264 | 0.022511323 |
| DDX3Y | 1.357024063 | 0.014123358 | 1.445006764 | 0.010260615 |
| RPS4Y1 | 1.364400491 | 0.006569692 | 1.339369328 | 0.007515011 |
| TIMP4 | 1.059485885 | 0.000160521 | 1.326405655 | 0.000102218 |
| PDK4 | 1.370332718 | 0.018510126 | 1.302479299 | 0.016500786 |
| UTY | 1.107806264 | 0.009645217 | 1.195295115 | 0.005700333 |
| USP9Y | 1.218318954 | 0.010853498 | 1.176296052 | 0.014883366 |
| KDM5D | 1.039923626 | 0.003426825 | 1.148707276 | 0.002398783 |
| TNFRSF12A | 1.040096615 | 1.10E-05 | 1.12750581 | 0.000368102 |
