# Supplementary material for: VCAM1 expression in the myocardium is associated with the risk of heart failure and immune cell infiltration in myocardium
Source: Sci Rep. 2021 Sep 30;11:19488. doi: 10.1038/s41598-021-98998-3 (PMC8484263; doi:10.1038/s41598-021-98998-3)
Supplement: Supplementary file 5 — Supplementary Information 5. [file 41598_2021_98998_MOESM5_ESM.docx]

|  | logFC | P.Value |
| --- | --- | --- |
| KEGG_PATHOGENIC_ESCHERICHIA_COLI_INFECTION | -95.56325114 | 0.001453022 |
| KEGG_AMYOTROPHIC_LATERAL_SCLEROSIS_ALS | -44.85435694 | 0.004862616 |
| KEGG_ALPHA_LINOLENIC_ACID_METABOLISM | -81.53809868 | 0.006363498 |
| KEGG_AMINO_SUGAR_AND_NUCLEOTIDE_SUGAR_METABOLISM | -78.36598291 | 0.006841812 |
| KEGG_PURINE_METABOLISM | -29.51187015 | 0.00742848 |
| KEGG_RIBOFLAVIN_METABOLISM | -58.34910709 | 0.00955531 |
| KEGG_N_GLYCAN_BIOSYNTHESIS | -41.54841641 | 0.010422038 |
| KEGG_BIOSYNTHESIS_OF_UNSATURATED_FATTY_ACIDS | -98.39778821 | 0.011985547 |
| KEGG_PRIMARY_BILE_ACID_BIOSYNTHESIS | 103.8257429 | 0.013901123 |
| KEGG_SPHINGOLIPID_METABOLISM | -69.35555127 | 0.016595809 |
| KEGG_LINOLEIC_ACID_METABOLISM | -54.02806191 | 0.01733008 |
| KEGG_STEROID_BIOSYNTHESIS | -99.0549491 | 0.020636114 |
| KEGG_GLUTATHIONE_METABOLISM | -51.62040775 | 0.022081708 |
| KEGG_GLYCOSAMINOGLYCAN_BIOSYNTHESIS_KERATAN_SULFATE | -65.34003489 | 0.028128498 |
| KEGG_SELENOAMINO_ACID_METABOLISM | -52.96726963 | 0.028656811 |
| KEGG_GLYCEROPHOSPHOLIPID_METABOLISM | -39.62157666 | 0.028977503 |
| KEGG_VEGF_SIGNALING_PATHWAY | -39.51499703 | 0.029423175 |
| KEGG_PENTOSE_PHOSPHATE_PATHWAY | -52.06985993 | 0.036762956 |
| KEGG_O_GLYCAN_BIOSYNTHESIS | -41.85800106 | 0.037372714 |
| KEGG_TASTE_TRANSDUCTION | 58.56847734 | 0.038965764 |
| KEGG_SNARE_INTERACTIONS_IN_VESICULAR_TRANSPORT | -32.04947395 | 0.0398779 |
| KEGG_UBIQUITIN_MEDIATED_PROTEOLYSIS | 17.11374405 | 0.041230882 |
| KEGG_CYSTEINE_AND_METHIONINE_METABOLISM | -27.45288637 | 0.041358303 |
| KEGG_APOPTOSIS | -46.70229223 | 0.043469164 |
| KEGG_WNT_SIGNALING_PATHWAY | 22.48682037 | 0.043975273 |
| KEGG_NON_HOMOLOGOUS_END_JOINING | -69.67770802 | 0.044697933 |
